# Comparison of efficacy and safety among four therapeutic regimens in primary membranous nephropathy with high anti-PLA2R antibody titers: a single-center retrospective cohort study

**DOI:** 10.1186/s12882-026-05327-9

**Published:** 2026-08-31

**Authors:** Yuxiu Huang, Xinxuan Meng, Shengqin Wu, Xia Miao, Lili Qin, Kunying Zhang, Jingshu Sun, Jianying Wang

**Affiliations:** 1 Nephrology Medical Center, Weifang People’s Hospital, Shandong Second Medical University, Weifang, Shandong 261000 China; 2 School of Clinical Medicine, Shandong Second Medical University, Weifang, Shandong 261053 China; 3 Clinical Laboratory, Weifang People’s Hospital, Shandong Second Medical University, Weifang, Shandong 261000 China

**Keywords:** Primary membranous nephropathy, Anti-PLA2R antibody level, Rituximab, Cyclosporine, Tacrolimus, Cyclophosphamide

## Abstract

**Background:**

Patients with primary membranous nephropathy (PMN) and high anti-PLA2R antibody titers (> 150 RU/mL) often respond poorly to conventional treatments, and the optimal immunosuppressive regimen remains unclear. We conducted a retrospective study to compare the efficacy and safety of four regimens in this high-risk population: rituximab monotherapy (RTX regimen), tacrolimus plus glucocorticoids (TAC regimen), rituximab plus cyclosporine (RTX plus CsA regimen), and cyclophosphamide plus glucocorticoids (CTX regimen).

**Methods:**

We retrospectively enrolled 185 patients with biopsy-proven PMN and anti-PLA2R titers > 150 RU/mL. All patients completed the full treatment course and the 24-month follow-up period. The primary outcome was total remission (TR) at 24 months, and the secondary outcomes included complete remission (CR), partial remission (PR), immunological remission (IR), relapse rate, time to remission, and adverse events.

**Results:**

At 24 months, the TR rates were 54.5% (24/44) for the RTX regimen, 52.2% (24/46) for the TAC regimen, 76% (38/50) for the RTX plus CsA regimen, and 73.3% (33/45) for the CTX regimen. Kaplan–Meier analysis revealed significant differences in cumulative IR rate (log-rank *P* = 0.0009), cumulative CR rate (log-rank *P* = 0.0072), and cumulative TR rate (log-rank *P* = 0.0005) among the four regimens. The TAC regimen had a significantly higher relapse rate (36.8%, 14/38) than the RTX plus CsA regimen (13.6%, 6/44) (log-rank *P* = 0.013).

**Conclusion:**

In this retrospective cohort, the RTX plus CsA regimen demonstrated durable efficacy, with a more favorable safety profile and a lower relapse rate. These preliminary findings support formal comparison of this regimen with current therapies in randomized controlled trials (RCTs).

**Supplementary Information:**

The online version contains supplementary material available at https://doi.org/10.1186/s12882-026-05327-9.

## Introduction

Primary membranous nephropathy (PMN) is the most common cause of nephrotic syndrome (NS) among adults [1]. Approximately 70%–80% of PMN cases are mediated by autoantibodies targeting the M-type phospholipase A2 receptor (PLA2R) expressed in podocytes [2]. Although approximately one-third of patients with PMN achieve spontaneous remission [3], long-term outcomes remain suboptimal, with frequent relapses and a marked association with progression to end-stage renal disease (ESRD) within 10 years [4]. This finding highlights the importance of durable therapies, particularly in high-risk patients.

The KDIGO guidelines [5] risk stratify patients to avoid unnecessary immunosuppression while targeting those at high or very high risk of progression. Patients with NS and anti-PLA2R antibody titers > 150 RU/mL are classified as high risk and require prompt immunosuppressive treatment. Anti-PLA2R antibodies are crucial biomarkers for predicting disease progression [6], treatment response [7], and prognosis[8–10].

Previous studies have reported that high anti-PLA2R antibody titers are associated with a progressive decline in kidney function [11] and lower rates of spontaneous remission [12, 13]. However, existing risk stratification models [14, 15] primarily rely on persistent proteinuria and short-term kidney function decline to predict chronic kidney disease (CKD) progression, and no model has comprehensively integrated all these clinical factors. Furthermore, clinical evidence guiding initial treatment selection based on anti-PLA2R antibody titers [16] remains insufficient.

Howman et al. [17] showed that alkylating agents are superior to cyclosporine (CsA) in slowing kidney function decline in patients with high-risk progressive MN. Rituximab (RTX) is an effective and safe alternative to alkylating agents [18, 19]. However, it induces a relatively slow treatment response and lower remission rates in the early stage [20, 21], particularly in patients with high anti-PLA2R antibody titers [22]. Therefore, high-risk patients may require an intensified RTX regimen or a combination with other immunosuppressive therapies to achieve favorable remission. Waldman et al. [23] reported that short-term CsA plus RTX therapy was effective in achieving both immunological and clinical remission in patients with high-risk PMN, providing a rationale for further study of this combination regimen.

To date, no study has directly compared these strategies in high-risk PMN patients with anti-PLA2R antibody titers (> 150 RU/mL). In this retrospective cohort study, we report the efficacy and safety of four regimens in this high-risk population: RTX monotherapy (RTX regimen), tacrolimus plus glucocorticoids (TAC regimen), RTX plus CsA regimen, and cyclophosphamide plus glucocorticoids (CTX regimen).

## Methods

### Study population

**Fig. 1:**
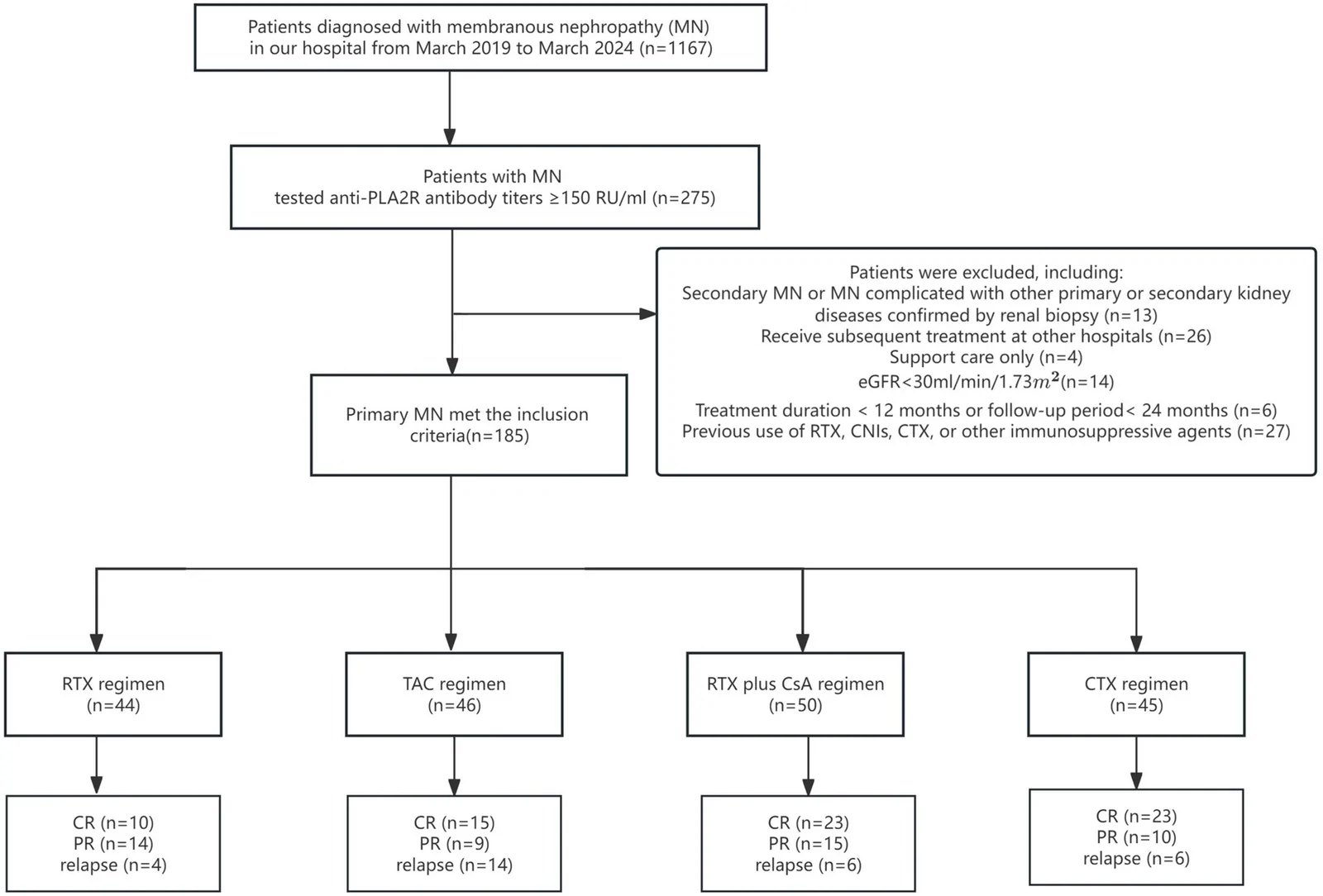
Flow chart of patient enrollment

This retrospective study was performed at the Kidney Disease Center of Weifang People’s Hospital. Between March 2019 and March 2024, 1167 patients clinically diagnosed with MN were admitted to our hospital. Of these patients, 275 had anti-PLA2R antibody titers > 150 RU/mL. Inclusion and exclusion criteria are shown in Fig. 1. A total of 185 adult patients with biopsy-proven PMN and anti-PLA2R antibody titers > 150 RU/mL were enrolled in the study. All the patients received standardized treatment at our hospital and completed the 24-month follow-up period.

### Eligibility criteria

Inclusion criteria: (1) Age ≥ 18 years; (2) Biopsy-proven PMN; (3) Clinical manifestations of NS with proteinuria > 3.5 g/24 h, serum albumin < 30 g/L and serum anti-PLA2R antibody titers > 150 RU/mL before treatment; (4) Initial treatment with one of the following four regimens: RTX monotherapy, TAC plus glucocorticoids (GCs), RTX plus CsA, or CTX plus GCs for approximately 12 months; (5) Complete follow-up data for ≥ 24 months.

Exclusion criteria: (1) Estimated glomerular filtration rate (eGFR) < 30 ml/min/1.73 m² (calculated using the CKD-EPI equation); (2) Previous use of RTX, calcineurin inhibitors (CNIs), cyclophosphamide (CTX) or other immunosuppressants; (3) Irregular medication use or incomplete follow-up records; (4) Secondary MN (including those associated with systemic lupus erythematosus, malignancy, hepatitis B or C virus infection, active HIV infection, diabetes mellitus, or severe infection) or comorbidity with other primary or secondary kidney diseases confirmed by renal biopsy.

### Treatment protocols

In this retrospective study, patients were categorized into four groups based on their immunosuppressive regimens used in real-world clinical practice: rituximab monotherapy (RTX regimen), tacrolimus plus GCs (TAC regimen), rituximab plus cyclosporine (RTX plus CsA regimen), and cyclophosphamide plus GCs (CTX regimen). The RTX regimen consisted of two intravenous doses of rituximab (1000 mg each) administered two weeks apart. During the 12-month treatment period, CD19 + B-cell counts were measured monthly until immunological remission (IR) was achieved and additional doses (1000 mg) of RTX were given when CD 19 + B-cell counts ≥ 5 cells/µL. After IR, anti-PLA2R antibody titers were regularly monitored. If the anti-PLA2R antibody titer increased (> 2 RU/mL), rituximab was re-administered. To reduce infusion-related reactions, patients received premedication protocols, including intravenous dexamethasone (10 mg), oral acetaminophen (0.5 g), and intramuscular promethazine (25 mg). The TAC regimen combined oral tacrolimus (TAC) initiated at 0.05–0.1 mg/kg/d (target trough concentration 3–8 ng/ml) with oral prednisone at 0.15–0.3 mg/kg/d. If IR was achieved, TAC was tapered gradually and therapy was maintained for at least 12 months. Otherwise, the full TAC dose was reduced after 12 months and discontinued after 6-month period. Prednisone was gradually tapered after 4–8 weeks and discontinued within 12 months. RTX was administered as a monotherapy regimen for the RTX plus CsA regimen. Oral CsA at 3.5 mg/ kg/d (targeting a trough of 125–225 ng/ml) was administered and tapered within 6 months after IR. TAC and CsA were administered in two equal doses at 12-hour intervals. The CTX regimen comprised cyclophosphamide and GCs. Patients received oral cyclophosphamide at 1.0-1.5 mg/kg/d for 6 months (cumulative dose < 0.2 g/kg), and oral prednisone 0.8-1.0 mg/kg/d for 4–8 weeks, with a maximum daily dose <60 mg/d, followed by a gradual taper over 10–12 months.

### Monitoring and follow-up protocol

All patients enrolled in this retrospective study completed the full treatment course and 24-month follow-up. A flowchart of the study enrollment is shown in Fig. 1. Laboratory assessments included routine blood and urine tests, liver and kidney function, blood lipids, 24-hour proteinuria quantification, Immunoglobulin G (IgG) and anti-PLA2R antibody titers, and circulating B-cell count. Monthly monitoring was performed for the first 3 months and quarterly thereafter. Monitoring of CD19 + B-cells is a good predictor of treatment response and can guide additional RTX dosing [24]. To evaluate the sufficiency of B-cell depletion, CD 19 + B-cell counts were measured after each RTX infusion and then monthly until IR. Anti-PLA2R antibody titers were measured using a standardized commercial enzyme-linked immunosorbent assay (ELISA). The follow-up data included treatment duration, response status, relapse rate, and adverse events.

### Treatment responses

The primary outcome was total remission (TR; complete or partial remission) at 24 months. Secondary outcomes included the rates of TR at 6 and 12 months; complete remission (CR), partial remission (PR), and immunological remission (IR) at 6,12,24 months, as well as relapse rate; time to CR, PR, and IR; and adverse events.

Clinical remission was assessed according to established criteria [25]. CR was defined as proteinuria < 0.3 g/24 h, serum albumin ≥ 35 g/L, and stable renal function. PR was defined as proteinuria 0.3–3.5 g/24 h and at least 50% reduction from baseline, serum albumin ≥ 30 g/L and stable renal function. IR was defined as anti-PLA2R antibody titers ≤ 2 RU/mL. The time to remission was defined as the interval from the first treatment to the achievement of PR, CR, or IR. B-cell depletion was defined as CD19 < 5 cells/µl. Relapse was defined as proteinuria>3.5 g/24 h in patients who previously achieved CR or PR. For the multivariate logistic regression analysis, we used a stricter composite endpoint termed “disease remission”. Disease remission was defined as the simultaneous achievement of clinical remission (CR or PR) and immunological remission (anti-PLA2R antibody < 2 RU/mL). Those who did not meet these dual criteria were classified as non-responders. This stricter definition was adopted because proteinuria remission alone may overestimate treatment efficacy, as recommended by recent studies [26].

## Supplementary content

### Ethics statement

This study was conducted in accordance with the Declaration of Helsinki and approved by the Ethics Committee of Weifang People’s Hospital (Approval No. KYLL20251126-7). All patients provided written informed consent prior to their inclusion in the study, in compliance with national legislation and institutional ethical guidelines.

### Statistical methods

Statistical analyses were performed using R software (version 4.3.2). Normally distributed continuous variables were presented as mean ± standard deviation (SD) and compared using one-way analysis of variance **(**ANOVA). Non-normally distributed continuous variables were described as medians (interquartile range [IQR]) and compared using the Kruskal-Wallis test. Categorical variables were presented as percentages and were analyzed using Pearson’s chi-square test. All P values were two-tailed, and *P* < 0.05 was considered statistically significant. The Kaplan-Meier method was used to estimate the cumulative remission rates across different treatment regimens. Adjusted logistic regression models were used to assess the effect of treatment regimens on clinical outcomes after controlling for covariates.

## Results

### General baseline parameter

A total of 185 patients with PMN were enrolled, with a mean age of 57.08 ± 12.31 years, of whom 116 (62.7%) were male. At baseline, mean proteinuria was 10.46 ± 5.46 g/24 h, serum albumin was 24.26 ± 5.06 g/L, serum creatinine was 69.72 ± 24.01 µmol/L, and eGFR was 95.07 ± 21.30 mL/min/1.73 m². All patients had high anti-PLA2R antibody titers (> 150 RU/mL), with a mean titer of 339.38 ± 195.41 RU/mL. The baseline characteristics were comparable across the four treatment regimens. (Table 1). Baseline renal biopsy characteristics including histological MN stag, degree of interstitial fibrosis and tubular atrophy (IFTA), and glomerular sclerosis proportion, were summarized in Supplementary Table S1. No significant differences in histological characteristics were observed across the four regimens.

**Table 1:** Baseline characteristics of patients with PMN included in this study

Characteristic	Total (*n* = 185)	RTX (*n* = 44)	TAC (*n* = 46)	RTX plus CsA (*n* = 50)	CTX (*n* = 45)	*P* value
Male sex (n%)	116/185(62.7)	26 / 44 (59.1)	29/ 46 (63)	29/ 50 (58.0)	32 / 45 (71.1)	0.556
Age (years)	57.08 ± 12.31	55.91 ± 14.52	56.09 ± 13.21	57.94 ± 10.92	58.29 ± 10.57	0.977
History of hypertension (n%)	97/185(52.4)	24/44(54.5)	23/46 (50.0)	25/50(50.0)	25/45(55.6)	0.923
History of diabetes (n%)	36/185 (19.5)	10/44 (22.7)	6/46 (13.0)	12/50 (24)	8/45 (17.8)	0.523
History of diuretic use (n%)	91/185 (49.2)	24/44 (54.4)	16/46(34.8)	29/50(58.0)	22/45(48.9)	0.118
History of statin use (n%)	134/185 (72.4)	30/44 (68.2)	35/46 (76.1)	34/50(68.0)	35/45(77.8)	0.606
History of ACEI/ARB use (n%)	111/185 (60.0)	28/44 (63.6)	28/46 (52.2)	28/50 (56.0)	31/45 (68.9)	0.357
Total protein (g/l)	48.46 ± 6.93	47.07 ± 6.69	49.93 ± 6.77	48.73 ± 6.91	48.02 ± 7.27	0.298
Albumin (g/l)	24.26 ± 5.06	23.33 ± 4.25	25.33 ± 5.93	23.49 ± 3.53	24.92 ± 6.04	0.161
Anti-PLA2R antibody titer (RU/mL)	339.38 ± 195.41	316.99 ± 171.32	335.38 ± 206.65	402.81 ± 240.48	294.90 ± 125.75	0.120
Proteinuria (g/24 h)	10.46 ± 5.46	11.93 ± 5.54	9.40 ± 4.89	10.21 ± 5.49	10.39 ± 5.76	0.140
G Immunoglobulin (g/l)	5.44 ± 1.75	5.20 ± 1.67	5.45 ± 2.06	5.91 ± 1.83	5.17 ± 1.31	0.128
Cholesterol (mmol/l)	8.60 ± 2.55	8.39 ± 1.98	8.97 ± 2.76	8.57 ± 2.90	8.47 ± 2.44	0.715
TG (mmol/l)	3.01 ± 2.18	2.61 ± 0.89	3.04 ± 2.49	3.46 ± 3.10	2.98 ± 1.36	0.630
LDL (mmol/l)	5.64 ± 2.34	5.71 ± 1.73	5.86 ± 2.53	5.84 ± 2.62	5.13 ± 2.32	0.333
serum creatinine (µmol/L)	69.72 ± 24.01	80.50 ± 39.96	66.71 ± 14.81	67.58 ± 16.15	64.66 ± 13.38	0.657
eGFR (ml/min/1.73 m²)	95.07 ± 21.30	88.65 ± 29.39	97.26 ± 19.02	94.48 ± 18.85	99.76 ± 14.74	0.278
Absolute values of CD19 (/µL)	302.02 ± 120.09	291.31 ± 69.55		311.44 ± 151.44		0.606

Values are presented as number (%), median (IQR) or mean ± SD

ACEI, Angiotensin-Converting Enzyme Inhibitor; ARB, Angiotensin II Receptor Blocker; Anti-PLA2R antibody titer, Anti-Phospholipase A2 Receptor; TG, triglyceride; LDL, low-density lipoprotein; Absolute values of CD19, Absolute count of CD19-positive B cells

**The estimated glomerular filtration rate (eGFR**,** ml/min/1.73 m**^**2**^**) was calculated using the** Chronic Kidney Disease Epidemiology Collaboration (**CKD-EPI) equation with creatinine**

### Treatment exposure

Dosing for patients treated with TAC or CTX was administered as detailed in the Methods section. For patients receiving RTX containing regimens, dosing was guided by B-cell counts and anti-PLA2R antibody levels. In the RTX mono-therapy regimen, the median cumulative RTX dose was 4 g (IQR 4,5), and 42/44 (95.5%) required additional infusions. In the RTX plus CsA regimen, the median cumulative RTX dose was 4 g (IQR 3,4), and 45/50 (90.0%) required additional infusions. Detailed RTX exposure data summarized in Supplementary Table S2.

### Efficacy assessment

The primary and secondary outcomes are shown in Table 2. For the primary outcome, 24-month TR rates were 54.4% (24/44) in the RTX regimen, 52.2% (24/46) in the TAC regimen, 76% (38/50) in the RTX plus CsA regimen, and 73.3% (33/45) in the CTX regimens. Compared with the TAC regimen, the RTX plus CsA regimen demonstrated a significantly higher TR rate at 24 months (76.0% vs. 52.2%, *P* = 0.015). For secondary outcomes, significant differences in TR (*P* = 0.003) and IR (*P* = 0.001) rates were observed among the four regimens as early as 6 months, and these differences remained at 24 months. At 6 months, the CTX regimen achieved the highest TR (46.7%) and IR (48.9%) rates, followed by the RTX plus CsA regimen (46.0% TR rate, 44.0% IR rate). The RTX regimen had the lowest remission rate among four regimens (13.6%). At 12 months, the CTX regimen showed the highest CR rate (37.8%; *P* = 0.006), whereas the RTX plus CsA regimen showed the highest TR (70.0%; *P* = 0.008) and IR (80.0%; *P* = 0.005) rates. At 24 months, the CTX regimen maintained the highest CR rate (51.1%, *P* = 0.023), followed by the RTX plus CsA regimen (46%). The RTX plus CsA regimen meanwhile maintained the highest TR (76%, *P* = 0.025) and IR (86%, *P* = 0.001) rates.

**Table 2:** Remission and relapse in patients

Characteristic	RTX (*n* = 44)	TAC (*n* = 46)	RTX plus CsA (*n* = 50)	CTX (*n* = 45)	*P* value
Complete remission(CR)					
6months (n%)	1 / 44 (2.3)	0/ 46 (0)	3/ 50 (6.0)	0 / 45 (0)	0.139
12 months (n%)	3 / 44 (6.8) *	11 /46 (23.9)	15 / 50 (30.0)	17 /45 (37.8)	0.006*
24 months (n%)	10 / 44 (22.7) *	15 /46 (32.6)	23 / 50 (46.0)	23 /45 (51.1)	0.023*
Partial remission (PR)					
6months (n%)	5 / 44 (11.4) *	18 /46 (23.9)	20 / 50 (40.0)	21 /45 (46.7)	0.002*
12 months (n%)	15 / 44 (34.1)	21 /46 (45.7)	20 / 50 (40.0)	14 /45 (31.1)	0.490
24 months (n%)	14 / 44 (31.8)	9 /46 (19.6)	15 / 50 (30.0)	10 /45 (22.2)	0.472
Total remission(TR)					
6months (n%)	6 / 44 (13.6) **	18 /46 (39.1)	23 / 50 (46.0)	21 /45 (46.7)	0.003*
12 months (n%)	18 / 44 (40.9) *	32 /46 (69.6)	35 / 50 (70.0)	31 /45 (68.9)	0.008*
24 months (n%)	24 / 44 (54.5) *	24 /46 (52.2) *	38 / 50 (76.0)	33 /45 (73.3)	0.025*
Immunological remission (IR)					
6months (n%)	6 / 44 (13.6) **	12 /46 (26.1)	22 / 50 (44.0)	22 /45 (48.9)	0.001*
12 months (n%)	24 / 44 (54.5) *	24 /46 (52.2) *	40 / 50 (80.0)	34 /45 (75.6)	0.005*
24 months (n%)	31 / 44 (70.5)	23 /46 (50.0) *	43 / 50 (86.0)	35 /45 (77.8)	0.001*
Time to immunological remission (mon)	10(7,14)	8(5,11)	7(4,10)	6(4.5,10)	
Time to complete remission (mon)	18(12,19)	12(11,19)	12(9,14)	11(10,14)	
Time to partial remission (mon)	12(7,17)	7(5,9)	6(4,9.5)	6(5,9)	
IR-PR	2.38 ± 4.85	-1.75 ± 5.03	0.52 ± 3.66	0.33 ± 3.57	
PR-CR	9.60 ± 3.50	7.40 ± 2.32	7.00 ± 3.49	6.92 ± 5.25	
Relapse (n%)	4/28(14.3)	14/38 (36.8) *	6/44(13.6)	6/39(15.4)	*P* = 0.031*
Cumulative administration dose (g)	4 (4,5)		4 (3,4)	12.50 ± 3.84	

*P < 0.05, **P < 0.001, RTX plus CNI regimens as control

**Fig. 2:**
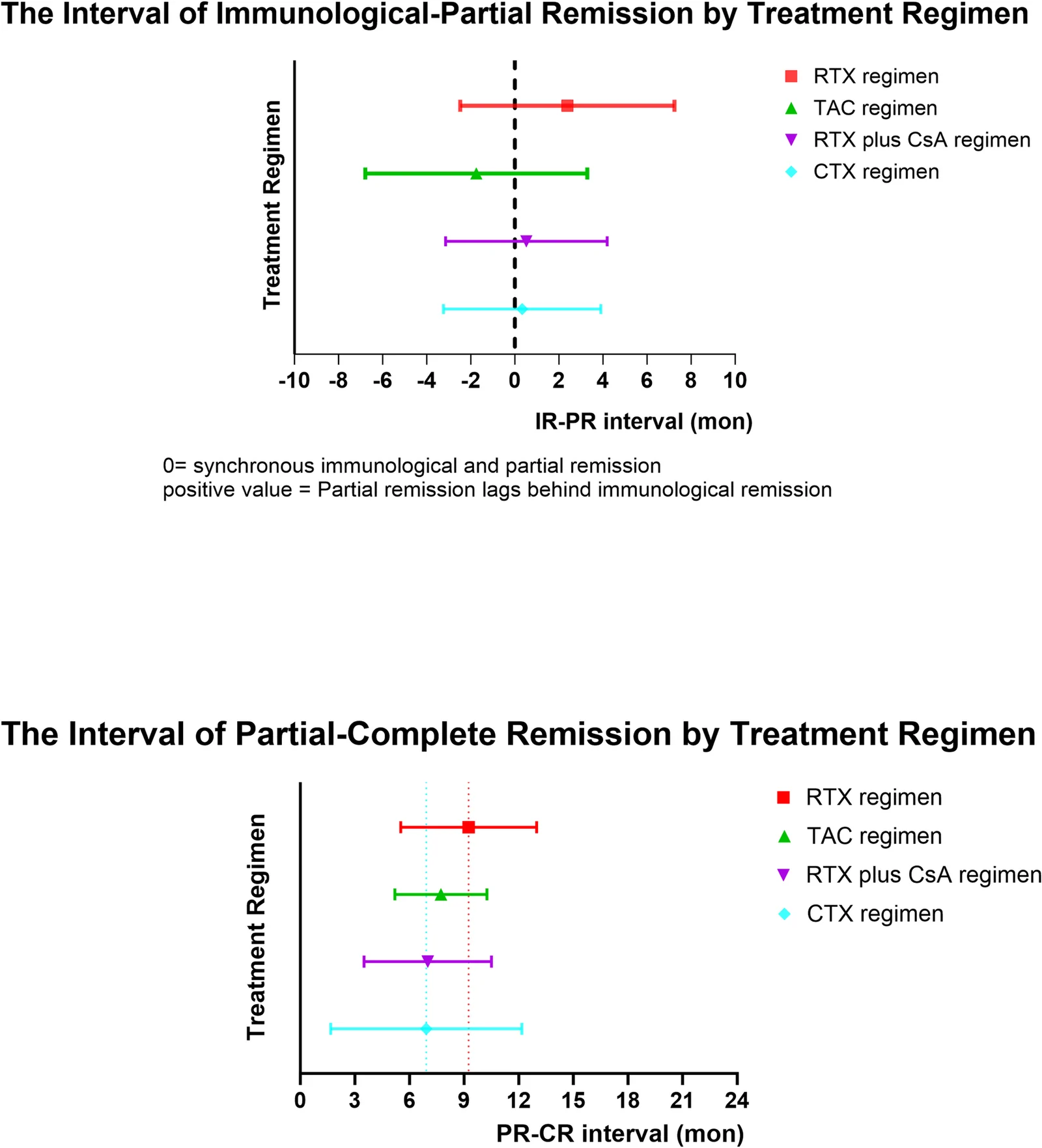
The interval of remission by treatment regimen

The CTX regimen achieved remission the most rapidly, followed by the RTX plus CsA regimen. The TAC regimen included 10 patients with clinical remission but no immunological remission, 4 of whom relapsed. The RTX regimen showed the longest time to PR [12 months (IQR 7, 17)] and IR [10 months (IQR 7, 14)]. We further calculated the time intervals from IR to PR and PR to CR (Fig. 2). The CTX regimen had the shortest time intervals from IR to PR (0.33 ± 3.57 months) and from PR to CR (6.92 ± 5.25 months), followed by the RTX plus CsA regimen (0.52 ± 3.66 months; 7.00 ± 3.49 months). For the TAC regimen, the time interval from IR to PR was − 1.75 ± 5.03 months. The RTX regimen had the longest mean intervals both from IR to PR (2.38 ± 4.85 months) and from PR to CR (9.60 ± 3.50 months) (Table 2; Fig. 2).

**Fig. 3:**
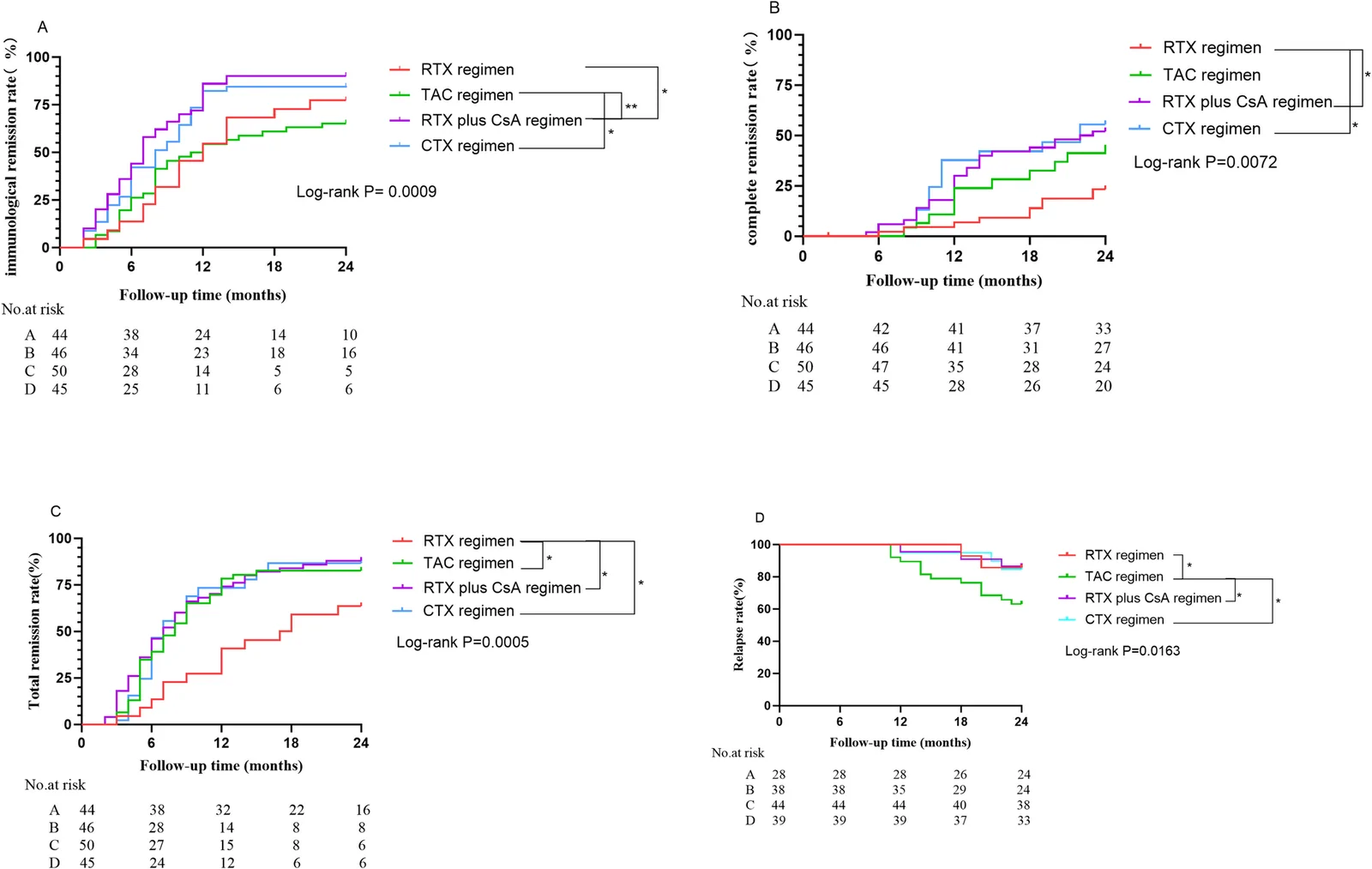
Kaplan-Meier analysis of (**A**) immunological remission rate, (**B**) complete remission rate, (**C**) total remission rate, (**D**) relapse rate in four regimens. *P < 0.05, **P < 0.001

During the 24-month follow-up period, Kaplan–Meier analysis showed significant differences in cumulative IR (log-rank *P* = 0.0009), CR (log-rank *P* = 0.0072), and TR rates (log-rank *P* = 0.0005) among the four regimens. Specifically, the RTX plus CsA regimen had a significantly higher cumulative IR rate than the TAC regimen (HR 2.277, 95% CI 1.398–3.711, Log-rank *P* = 0.001) and a higher cumulative TR rate than the RTX regimen (HR 2.518, 95% CI 1.374–4.613, Log-rank *P* < 0.0001). The CTX regimen achieved a higher cumulative CR rate than the RTX regimen (HR 3.091, 95% CI 1.576–5.97, Log-rank *P* = 0.0012), with no difference from the RTX plus CsA regimen (*P* = 0.689). Relapse was analyzed in 149 patients who achieved clinical remission during the entire follow-up period, and the cumulative relapse rate differed significantly (log-rank, test *P* = 0.0163). Notably, **t**he TAC regimen showed the highest rate, which was significantly higher than the RTX plus CsA regimen (HR 3.120,95% CI 1.272–7.658, log-rank *P* = 0.013). (Fig. 3). Subgroup analyses stratified by baseline anti-PLA2R antibody titers [extremely high (> 500RU/mL) vs. moderately high (150–500 RU /mL)] are summarized in Supplementary Table S3.

### Changes in laboratory indexes

**Fig. 4:**
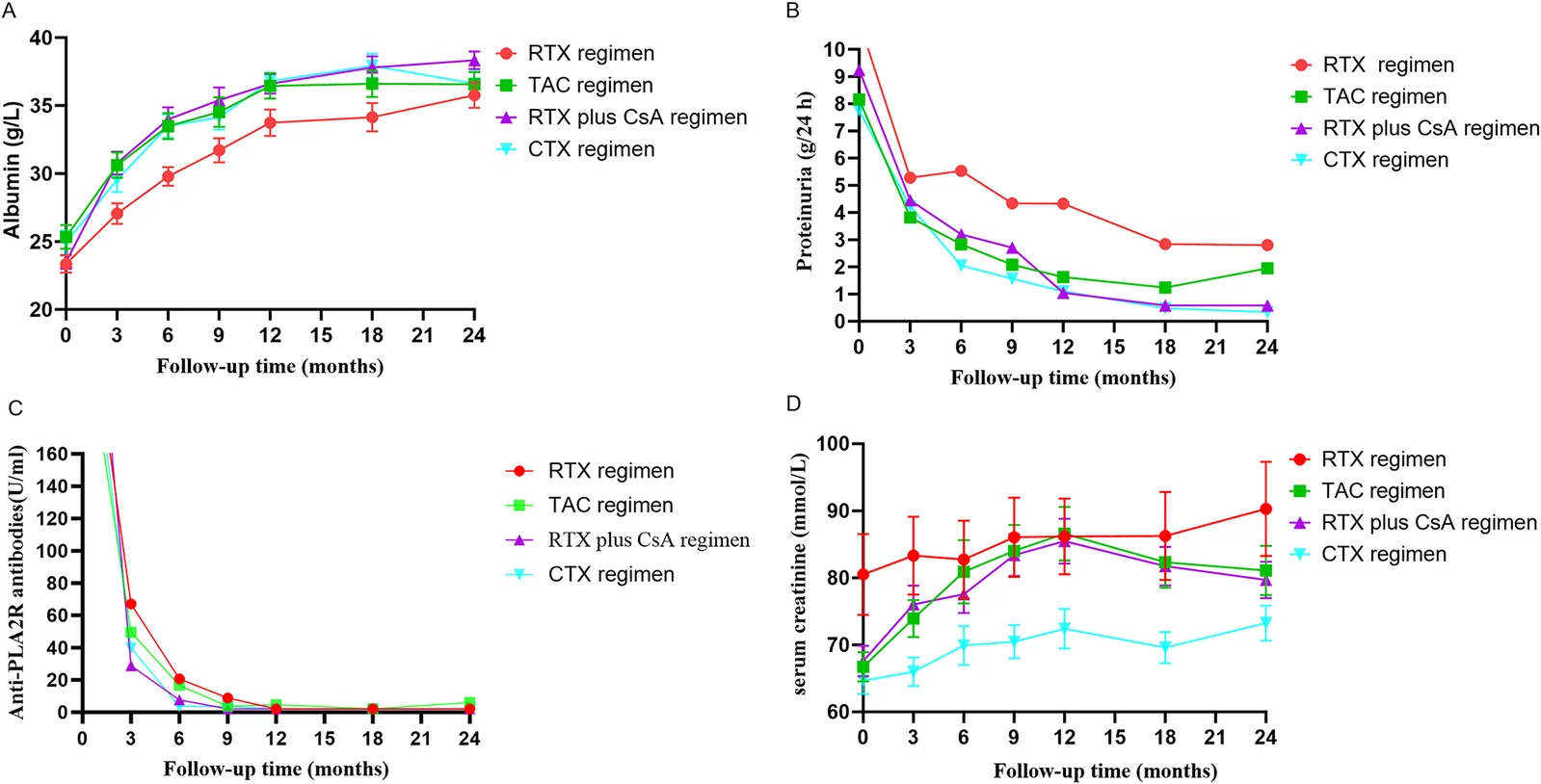
Serial levels of (**A**) albumin, (**B**) proteinuria, (**C**)anti-PLA2R antibody, (**D**) serum creatinine across the four regimens in patients who had been followed up for 24 months. Data are presented as the medians (IQR) over time (**B**, **C**) or mean ± SD (**A**, **D**)

During the follow-up period, all PMN patients showed an overall decreasing trend in anti-PLA2R antibody titers and proteinuria and an upward trend in serum albumin (Fig. 4). At 24 months, serum albumin increased from 24.26 ± 5.06 g/L to 36.86 ± 6.01 g/L (*P* > 0.05). At 12 months, the RTX regimen resulted in lower albumin levels than the other regimens (*P* = 0.022). Anti-PLA2R antibody titers decreased from 339.38 ± 195.41 RU/mL to 2 RU/mL (IQR 2, 18.40) at 12 months and 2 RU/mL (2, 16.68) at 24 months. Statistically significant differences in anti-PLA2R antibody titers were observed among the four regimens at both 12 months (*P* = 0.02) and 24 months (*P* = 0.003). Both the CTX and RTX plus CsA regimens reduced antibody titers to 2 RU/mL (IQR 2, 2) by 12 months, with the overall antibody titers becoming negative. Proteinuria decreased from 10.46 ± 5.46 g/24 h to 2.08 g/24 h (IQR 0.40, 4.37) at 12 months and 1.19 g/24 h (IQR 0.20, 4.27) at 24 months, with statistically significant differences in proteinuria among the four regimens at both 12 months and 24 months (*P* = 0.001). Compared to the RTX regimen, the RTX plus CsA regimen exhibited lower proteinuria levels at both 12 months [4.32 g/24 h (IQR 2.08,7.66) vs. 1.05 g/24 h (IQR 0.27,3.83) *P* = 0.001] and 24 months [2.80 g/24 h (IQR 1.19,5.30) vs. 0.58 g/24 h (IQR 0.16,1.73) *P* = 0.001]. For renal endpoints, the CNI-based regimens resulted in transient serum creatinine levels. Specifically, the elevation began at 6 months and peaked at 12 months, with significant differences in serum creatinine level (*P* = 0.011) and eGFR (*P* = 0.046) at 12 months. However, none of the patients progressed to ESRD. By 24 months, serum creatinine levels in the CNI-based regimens had normalized, with no significant differences among the four regimens (*P* > 0.05) (Table 3).

**Table 3:** Clinical characteristics of PMN patients after treatment for 12 months and 24 months

Characteristic	Total (n=185)	RTX (n = 44)	TAC (n=46)	RTX plus CsA (n = 50)	CTX (n = 45)	P value
**At 12 months**						
Albumin (g/L)	35.93±5.62	33.73 ± 6.42	36.45 ± 6.32	36.60 ± 5.08	36.81 ± 3.98	0.022*
Anti-PLA2R antibody titer (RU/mL)	**2(2,18.40)**	**2 (2, 29.79)**	4.74 (2, 40.79) *	2 (2, 2)	2 (2, 2)	0.020*
Proteinuria (g/24h)	2.08 (0.40, 4.37)	4.32 (2.08, 7.66)	1.62 (0.30, 3.79)	1.05 (0.27, 3.83)	1.10 (0.32, 3.64)	0.001*
serum creatinine (μmol/ L)	82.74±28.03	86.18 ± 37.54	86.58 ± 27.10	85.48 ± 23.64	72.40 ± 19.75	0.011*
eGFR (ml/min/1.73 m²)	84.46±24.94	83.80±29.14	81.73±25.82 *	80.14±23.27 *	92.70±19.68	0.046*
**At 24 months**						
Albumin (g/L)	36.86±6.01	35.76 ± 6.21	36.56 ± 6.09	38.33 ± 4.57	36.61 ± 6.93	0.116
Anti-PLA2R antibody titer (RU/mL)	2 (2,16.68)	2 (2, 64.43)	3.37 (2, 40.79) *	2 (2, 2)	2 (2, 4.74)	0.003*
Proteinuria (g/24h)	**1.19 (0.2, 4.27)**	2.80 (1.19,5.30) *	1.95 (0.25, 4.84)	0.58 (0.16, 1.73)	0.34 (0.11, 3.27)	0.001*
serum creatinine (μmol/ L)	81.52±31.56	92.54 ± 51.80	81.08 ± 24.88	79.68 ± 19.16	73.24 ± 17.48	0.245
eGFR (ml/min/1.73 m²)	86.12±22.95	82.38±29.23	86.11±22.18	84.65±20.21	91.42±19.08	0.298

We divided the patients with 12-month PR into three subgroups based on their clinical outcomes at 24 months (progression to CR, maintenance of PR, or disease relapse). Anti-PLA2R antibody positivity rate differed significantly between the subgroups (*P* = 0.002) (Table 4). Although the differences in treatment regimens were not significant (*P* = 0.302), the CTX regimen had the highest CR rate (57.1%) and lowest relapse rate (14.3%), followed by RTX plus CsA regimen (45.0% CR; 5.0% relapse). The TAC regimen and RTX had highest relapse rates (28.6% and 26.7%, respectively).

### Logical analysis of the correlation between treatment regimens and treatment response

The clinical characteristics of responders and non-responders are summarized in Table 5. Of the 185 patients, 135 were responders (meeting the above disease remission criteria) and 50 were non-responders. Non-responders had higher anti-PLA2R antibody titers (*P* = 0.001), higher proteinuria levels (*P* = 0.001), and lower IgG levels (*P* = 0.001) (Table 5). Univariate logistic regression analysis showed that treatment regimen was associated with treatment response. Using RTX plus CsA regimen as a reference, odds ratio (OR) was 0.275 for the TAC regimen (*P* = 0.009) and 0.296 for the RTX regimen(*P* = 0.013). After multivariate adjustment for covariates (including IgG, proteinuria, and anti-PLA2R antibody titer), treatment regimen remained an independent associated factor. The OR were 0.141 for the RTX regimen (*P* = 0.003), 0.176 for the TAC (*P* = 0.008), and 0.893 for the CTX regimen (*P* = 0.875) (Table 6), respectively.

**Table 4:** The heterogeneity and long-term prognosis of patients with PR at 12 months

Characteristic \ Clinical Endpoints	CR (*n* = 30)	PR (*n* = 27)	relapse (*n* = 13)	
Anti-PLA2R antibody positivity, n (%)	2/ 30 (6.7)	6/27 (22.2)	7/13 (53.8) *	P = 0.002 *
Serum Albumin ≥ 35 g/L, n (%)	25/30 (83.3)	20/27 (74.1)	8/13 (61.5)	P = 0.300
eGFR (ml/min/1.73 m²)				P = 0.653
≥ 90	16 / 30(53.3)	12/27 (44.4)	8 / 13 (61.5)	
60–90	10/30(33.3)	10/27(37.0)	2 / 13 (15.4)	
30–60	4/30(13.3)	5/27(18.5)	3 / 13 (23.1)	
**Treatment regimen (n%)**				P = 0.302
RTX regimen (*n* = 15)	7 / 15 (46.7)	4 / 15 (26.7)	4 / 15 (26.7)	
CNI regimen (*n* = 21)	6 / 21 (28.6)	9 / 21 (42.9)	6 / 21 (28.6)	
RTX plus CNI regimen (*n* = 20)	9 / 20 (45.0)	10 / 20 (50.0)	1 / 20 (5.0)	
CTX regimen (*n* = 14)	8 / 14 (57.1)	4 / 14 (28.6)	2/14 (14.3)	

**Table 5:** Baseline characteristics in patients with different response to treatment

Characteristic	Responders (*n* = 135)	Non-responders (*n* = 50)	*P* value
Male sex (n%)	82/ 135 (60.7)	34 / 50 (68.0)	0.365
Age (years)	56.40 ± 12.20	58.92 ± 12.55	0.132
History of hypertension (n%)	66/135 (48.9)	31/50 (62.0)	0.113
History of diabetes (n%)	26/135 (19.3)	10/50 (20.0)	0.910
History of diuretic use (n%)	71/135 (52.6)	20/50 (40.0)	0.128
History of statin use (n%)	101/135 (74.8)	33/50 (66.0)	0.731
History of ACEI/ARB use (n%)	84/135 (62.2)	27/50 (54.0)	0.311
Total protein (g/L)	48.99 ± 6.72	47.03 ± 7.35	0.087
Albumin (g/L)	24.81 ± 4.97	23.08 ± 5.74	0.054
**Anti-PLA2R antibody titer (RU/mL)**	**304.98 ± 150.53**	**432.27 ± 263.30**	0.001*
**Proteinuria (g/24h)**	**9.31 ± 4.32**	**13.58 ± 6.88**	0.001*
**G Immunoglobulin (g/L)**	**5.77 ± 1.80**	**4.58 ± 1.30**	0.001*
Cholesterol (mmol/L)	8.54 ± 2.49	8.78 ± 2.73	0.865
TG (mmol/L)	3.02 ± 2.20	2.98 ± 2.15	0.891
LDL (mmol/L)	5.61 ± 2.24	5.72 ± 2.60	0.781
serum creatinine (µmol/L)	68.68 ± 24.16	72.54 ± 23.60	0.129
eGFR (ml/min/1.73 m²)	96.62 ± 20.67	90.93 ± 23.48	0.224
**Treatment regimen (n%)**			0.002*
**RTX regimen**	**26 / 135 (19.3)**	**18 / 50 (36.0)**	
**CNI regimen**	**28 / 135 (20.7)**	**18 / 50 (36.0)**	
**RTX plus CNI regimen**	**42 / 135 (31.1)**	**8 / 50 (16.0)**	
**CTX regimen**	**39 / 135 (28.9)**	**6 / 50 (12.0)**	

### Adverse events

All adverse events (AEs) are detailed in Table 7. Overall, 24.7% (46/185) of patients experienced at least one AE, with incidence rates of 15.9% (7/44; RTX regimen), 26.1% (12/46; TAC regimen), 18% (9/50; RTX plus CsA regimen), and 37.7% (17/45; CTX regimen). Infusion-related reactions are most common with RTX-based regimens, which are mild and easily manageable. Elevated serum creatinine levels occurred more frequently in patients without ESRD receiving CNI-based regimens during follow-up. Gastrointestinal events and hyperglycemia were more common in the TAC and CTX regimens. The incidence of leukopenia was significantly higher in the CTX regimen (8.89%). No malignancies were reported.

**Table 6:** Univariate and multivariate logistic regression for the risk factors of response

	Univariate logistic regression		Multivariate logistic regression	
Variable	OR (95% CI)	P	OR (95% CI)	P
**Treatment regimen**				
RTX plus CsA regimen (reference)	1		1	
RTX regimen	**0.296 (0.113, 0.774)**	0.013*	**0.141 (0.039, 0.502)**	0.003*
TAC regimen	**0.275 (0.105, 0.723)**	0.009*	**0.176 (0.049, 0.635)**	0.008*
CTX regimen	1.238 (0.394, 3.890)	0.715	0.893 (0.218, 3.658)	0.875
Male sex (n%)	1.373 (0.691, 2.731)	0.365		
Age (years)	0.983 (0.956, 1.010)	0.217		
Total protein (g/L)	1.043 (0.994,1.094)	0.089		
Albumin (g/L)	1.069 (0.998, 1.145)	0.056		
Anti-PLA2R antibody titer (RU/mL)	**0.997 (0.995, 0.999)**	0.001*	**0.996 (0.994, 0.998)**	0.001*
Proteinuria (g/24h)	**0.868 (0.815, 0.925)**	0.001*	**0.888 (0.825, 0.957)**	0.002*
G Immunoglobulin (g/L)	**1.627 (1.275, 2.076)**	0.001*	1.558 (1.176, 2.064)	0.002*
Cholesterol (mmol/L)	0.963 (0.850,1.092)	0.558		
TG (mmol/L)	1.009 (0.867, 1.173)	0.910		
LDL (mmol/L)	0.981 (0.855,1.126)	0.789		
serum creatinine (μmol/L)	0.994 (0.981, 1.007)	0.336		
eGFR (ml/min/1.73 m²)	1.012 (0.997,1.027)	0.113		

The treatment regimen was a multicategorical variable, with RTX plus CsA as the reference regimen, and immunoglobulin, anti-PLA2R antibody titer, proteinuria, and cholesterol were continuous variables

## Discussion

PMN patients with anti-PLA2R antibody titers > 150 RU/mL are at high risk of poor treatment response, frequent relapse, and progressive kidney function decline. Although the current guidelines [5] recommend several immunosuppressive regimens, direct head-to-head comparisons in this high-titer subgroup remain rare. In this study, we compared the efficacy and safety of four immunosuppressive regimens in patients with anti-PLA2R antibody titers > 150 RU/mL.

**Table 7:** Adverse events according to treatment group

Events	RTX (n = 44)	TAC (n=46)	RTX plus CsA (n = 50)	CTX (n = 45)
All adverse event	7	16	11	24
Serious adverse event	2	1	0	3
Fatal	0	0	0	0
Nonfatal	2	1	0	3
Severe pneumoniae	2	1	0	3
End-stage renal disease	0	0	0	0
Cancer	0	0	0	0
Nonserious adverse events	5	15	11	21
Leukopenia	0	0	0	4
Other infections event	0	0	1	0
Herpes zoster	1	0	1	1
Infusion-related reactions	3	0	3	0
Hyperglycemia	0	4	0	7
Osteoporosis	1	0	0	1
Increased creatinine level	0	5	4	0
Gastrointestinal symptom	0	4	0	4
Hepatic dysfunction	0	2	2	4

Infusion-related reactions include rashes, erythema, pruritus, runny nose, and irritability

CTX plus GCs is a preferred first-line treatment for high-risk PMN. Previous studies [27] reported 12-month TR rates of 50%–60% with CTX therapy. The STARMEN and RI-CYCLO trials achieved more favorable outcomes, with 12-month TR rates of approximately 70% [28, 29]. However, these studies did not specifically focus on patients with anti-PLA2R antibody titers > 150 RU/mL. In our study, the CTX regimen induced rapid and effective remission, with the highest immunological (48.9%) and clinical remission (46.7%) rates at 6 months, and a favorable 12-month clinical remission rate (68.9%). The relapse rate was relatively low (15.4%) and the TR rate increased to 73.3% at 24 months, with the highest CR rate (51.1%; *P* = 0.023). These findings suggest that CTX remains effective in inducing remission in patients with high anti-PLA2R antibody titers. However, CTX-related toxicity remains a major limitation of this study. The cumulative CTX dose in our cohort was 12.50 ± 3.84 g, and the incidence of adverse events was higher compared with the other regimens (37.7% vs. 15.9%, 26.1%, and 18.0% for the RTX, TAC, and RTX plus CsA regimens, respectively).

CNIs plus GCs are commonly used as alternatives for patients who are intolerant of or have contraindications to CTX. Although CsA and TAC effectively induce short-term proteinuria remission [21, 30, 31], their major limitation is a high relapse rate of 40%–60% after discontinuation [32, 33]. In the MENTOR study [21], CsA achieved favorable early remission in patients with high anti-PLA2R antibody titers, but > 60% relapsed by 24 months after CsA discontinuation, leading to a poor 24-month TR rate of only 20%. In our cohort, the TAC regimen achieved a higher 12-month TR rate of 69.6% than reported in the MENTOR study, likely due to synergistic effects of TAC and GCs. The TAC plus GCs regimen exhibited superior efficacy in proteinuria remission compared with CNI monotherapy regimen [34]. Specifically, GCs reduce T lymphocyte activity, suppress B cell antibody responses, and act synergistically with CNIs to reduce proteinuria [35, 36]. However, consistent with previous studies [37], the TAC regimen showed a high relapse rate (36.8%) and a significantly higher cumulative relapse rate in survival analysis (log-rank *P* = 0.0163). Consequently, the 24-month remission rate declined to 52.2%. Patients who experience relapse often show a slow antibody decline or fail to achieve immunological remission. Thus, the TAC regimen may not be optimal for high-risk PMN patients, particularly for long-term remission maintenance.

RTX is an anti-CD20 monoclonal antibody that selectively depletes CD20 + B cells. Considering the disease course of MN, RTX has emerged as an ideal first-line agent, with adequate long-term follow-up [21]. However, patients with high anti-PLA2R antibody titers show a poor response to a standard-dose RTX regimen [20, 38]. Van de Logt et al. [39] found that standard-dose RTX was less effective than CTX in inducing IR in this subgroup, supporting the need for intensified dosing. Further studies [40] have shown that a second treatment course can improve RTX efficacy, and Sachin Naik et al. [41] reported that a cumulative RTX dose of 3 g achieved a response rate of at least 55% in high-titer PMN. In our study, we administered additional doses of RTX to 42/44 (95.5%) patients to maintain B-cell depletion. Nevertheless, RTX monotherapy resulted in a delayed response, with slow achievement of immunological and clinical remission, as we previously reported [42]. This may be attributable to long-lived memory plasma cells, which drive persistent auto antibody production, leading to treatment resistance and relapse [43, 44]. Despite its favorable safety profile and low relapse rate, RTX showed limited efficacy in this high-titer population.

Sequential therapy aims to alleviate proteinuria using CNIs and subsequently administer RTX to reduce the likelihood of relapse following CNI withdrawal. Sequential CNI followed by RTX has recently gained attention for high-risk PMN, with emerging evidence [45, 46] supporting its efficacy and safety. However, the STARMEN trial [29] showed that sequential treatment with TAC followed by RTX was inferior to the standard cyclical CTX therapy. Waldman et al. [23] reported that short-term CsA plus RTX treatment induces rapid and sustained remission in high-risk PMN. Our study extends these findings to patients with high anti-PLA2R antibody titers. CsA was selected as the CNI in our combination regimen because of its comparable efficacy, favorable safety, and superior cost-effectiveness to TAC in our clinical practice. In our cohort, RTX plus CsA achieved the highest 24-month TR rate (76%) and IR rate (86%), with a low relapse rate (13.6%) and a favorable safety profile. These findings support that RTX plus CsA may be a valuable treatment approach for high-risk PMN.

In the subgroup analysis stratified by baseline anti-PLA2R antibody titer, the RTX plus CsA regimen achieved the highest 24-month TR rate (83.8%) in patients with moderately high titers (150-500 RU/mL). In the extremely high titer subgroup (> 500 RU/mL), CTX demonstrated the numerically highest remission rate, followed by the combination regimen. Given the small sample size in the extremely high titer subgroup, these results should be interpreted with caution.

Notably, the composite endpoint of disease remission used in our regression analysis is stricter than conventional proteinuria-based remission. Incomplete clearance of anti-PLA2R antibodies has been associated with a higher risk of subsequent relapse [8], and recent recommendations [26] advocate incorporating immunological remission alongside proteinuria remission in the definition of disease remission in PMN. Using this composite endpoint, multivariate logistic analysis showed that RTX plus CsA regimen was independently associated with more favorable treatment responses compared to the TAC regimen or the RTX regimen.

Our findings challenge the traditional 6-month observation period for high-risk PMN. The low IR rates observed with RTX and TAC regimens at 6 months (13.6% and 26.1%, respectively) raise the question of whether earlier or more intensive intervention is warranted in this population. To understand the mechanisms underlying the different response patterns observed in our cohort, we analyzed remission timing, specifically the time intervals from IR to PR and PR to CR (Fig. 2). Consistent with previous studies [20], the CTX regimen achieved the shortest time to remission, as well as the shortest intervals from IR to PR and from PR to CR. This may be attributed to its immunomodulatory effects that disrupt DNA replication and cell division in T, B, and plasma cells [20]. The TAC regimen induces PR earlier than IR, which may be explained by the ability of CNIs to inhibit T-cell activation and stabilize the podocyte cytoskeleton, thereby rapidly reducing proteinuria [47]. This asynchrony between clinical and immunological remission is a key reason for high relapse rates. The RTX regimen exhibited a delayed onset of action, which was mainly attributed to increased RTX loss due to heavy proteinuria [48] and the formation of anti-rituximab antibodies in patients with high antibody titers [49]. Notably, the RTX plus CsA regimen exhibited an onset of action similar to that of the CTX regimen and shortened both time intervals. This may be explained by the fact that CNIs inhibit T-cell activation and reduce the urinary loss of RTX through their antiproteinuric effect mediated by vasoconstriction [50], while RTX depletes CD20 + B cells and decreases antibody production.

To assess the impact of regimen choice on long-term prognosis, we stratified patients who had achieved PR at 12 months into three subgroups according to their 24-month clinical outcomes (progression to CR, maintenance of PR, or disease relapse) (Table 4). We found that persistent anti-PLA2R antibody positivity during clinical remission was strongly associated with subsequent relapse (*P* = 0.002). This finding suggests that clinical remission alone may overestimate disease control, and highlights the potential value of regular antibody monitoring in patients at high risk of relapse [16]. For the CTX regimen, the majority of patients had achieved IR by 12 months, and durable remission was observed at 24 months (57.1% CR; 28.6% PR). These results support planned treatment cessation at 12 months in patients who have achieved IR. The RTX plus CsA regimen also achieved favorable long-term clinical remission (45% CR; 50% PR) and low relapse (5%), allowing discontinuation at 12 months with optional re-treatment upon antibody rebound. In contrast, the RTX monotherapy regimen showed a slow immunological response (median time of 10 months [IQR 7–14]) and required prolonged B-cell depletion to maintain remission. This is because B-cell reconstitution may induce disease relapse, making sustained depletion until IR is achieved essential. Given its limited efficacy in high-risk patients, RTX may be better utilized in combination with other agents. The TAC regimen was characterized by a low CR rate (28.6%), frequent relapse (28.6%), and failure to induce late immunological remission. Among the 6 relapsed patients in the TAC regimen, 2 failed to achieve IR during the follow-up period. Despite the limitations of a retrospective design, our data still show that IR is the cornerstone of durable disease control. Therefore, for high-risk patients who achieve proteinuria remission but have persistently positive anti-PLA2R antibody titers during the CNI tapering, the addition of RTX may be considered [51].

The most concerning aspect of immunosuppressive therapy is its adverse effects (AEs) (Table 7). The CTX regimen was associated with the highest incidence of AEs (37.7%), particularly gastrointestinal symptoms, hyperglycemia, and leukopenia. Given its significant toxicity, CTX should be used with caution in high-risk patients. Hyperglycemia was also common in the TAC regimen, potentially related to the adverse effects of GCs. The CNI-based regimens led to a transient decline in renal function during the induction phase, although none of the patients progressed to ESRD in our study. However, prolonged CNI exposure remains a concern because of its well-documented nephrotoxicity [52]. RTX-based regimens commonly cause mild transient infusion reactions, which resolve without complications. AEs with the RTX plus CsA combination regimen were comparable to those observed with RTX or TAC regimens. Importantly, the combination regimen achieved earlier immunological remission [median 7 months (IQR 4–10)]. CsA was gradually discontinued after IR was achieved, which reduced cumulative CNI exposure and was associated with fewer CNI-related adverse events and NS complications. Additionally, in the combination regimen, the cumulative RTX dose was lower than that in the RTX monotherapy regimen [4 g (IQR 3–4) vs. 4 g (IQR 4–5)], reducing the economic burden of long-term treatment. These data further support the safety profile of the RTX plus CsA regimen in high-risk PMN patients with baseline anti-PLA2R antibody titers > 150 RU/mL.

Currently, few studies have directly compared these four regimens. Our study provides novel insights by focusing on high-risk PMN patients with baseline anti‑PLA2R antibody titers > 150 RU/mL. Additionally, the observation period was extended to 24 months, which enabled a comprehensive assessment of treatment durability and relapse risk. However, this study has several limitations. First, as a single-center retrospective study with a relatively small sample size, the statistical power for subgroup analyses of clinical efficacy and adverse events was limited. Second, owing to the non-randomized design, treatment decisions were largely driven by objective clinical constraints, including patients’ financial burden, the availability of each regimen in routine clinical practice, and individual contraindications or tolerance. Although we attempted to standardize inclusion criteria and control for confounding, the possibility of residual selection bias may not be fully ruled out. Consequently, residual confounding may have influenced the observed differences in remission rates and relapse risk, and the findings should be interpreted with caution. Therefore, our conclusions require verification in large-scale randomized controlled trials (RCTs). Third, the combination regimen only used CsA. Thus the optimal CNI agent for RTX combination therapy remains unclear and requires further head-to-head comparisons in future studies.

In conclusion, our study showed that the RTX plus CsA regimen achieved long-term clinical and immunological remission rates comparable to those of the CTX regimen, with a more favorable safety profile and lower relapse rate. These findings suggest that it could serve as a promising therapeutic option for patients with PMN with high anti-PLA2R antibody titers (> 150 RU/mL), warranting further investigation in RCTs.

## Supplementary Information

Below is the link to the electronic supplementary material.

Supplementary Material 1

## Data Availability

The datasets used and/or analysed during the current study are available from the corresponding author on reasonable request.

## Abbreviations

AEs: Adverse effects
CKD: Chronic kidney disease
CNI: Calcineurin inhibitor
CR: Complete remission
CsA: Cyclosporine A
CT: Computed tomography
CTX: Cyclophosphamide
ESRD: End-stage renal disease
ELISA: Enzyme-linked immunosorbent assay
IFTA: Interstitial Fibrosis and Tubular Atrophy
IQR: Interquartile range
KDIGO: Kidney Disease: Improving Global Outcomes
LDL: Low-Density Lipoprotein
NS: Nephrotic Syndrome
PMN: Primary membranous nephropathy
PR: Partial remission
PLA2R: Phospholipase A2 receptor
RCT: Randomized controlled trial
RTX: Rituximab
TG: Triglycerides
TR: Total remission
