# Supplementary material for: Comparison of efficacy and safety among four therapeutic regimens in primary membranous nephropathy with high anti-PLA2R antibody titers: a single-center retrospective cohort study
Source: BMC Nephrol. 2026 Aug 31;27:515. doi: 10.1186/s12882-026-05327-9 (PMC13540925; doi:10.1186/s12882-026-05327-9)
Supplement: Supplementary file 1 [file 12882_2026_5327_MOESM1_ESM.docx]

**Supplementary Table S1. Baseline renal biopsy characteristics**

| Characteristic | RTX (n =44) | TAC (n=46) | RTX plus CsA (n = 50) | CTX (n=45) | P value |
| --- | --- | --- | --- | --- | --- |
| **MN histological stage,n(%)** |  |  |  |  | 0.368 |
| Stage 1 | 25/44(56.8%) | 29/46(63%) | 30/50(60%) | 31/45 (68.9%) |  |
| Stage I-II | 8/44(18.2%) | 12/46(26.1%) | 6/50(12%) | 7/45(15.6%) |  |
| Stage II | 7/44(15.9%) | 4/46 (8.75%) | 11/50(22%) | 5/45(11.1%) |  |
| Stage II-III | 4/44 (9.1%) | 1/46 (2.2%) | 3/50(6%) | 1/45(2.2%) |  |
| Stage III | 0 | 0 | 0 | 1(2.2%) |  |
| **IFTA grade,n(%)** |  |  |  |  | 0.807 |
| Grade 0 (no atrophy/fibrosis) | 18/44(40.9%) | 22/46(47.8%) | 23/50(46%) | 23/45(51.1%) |  |
| Grade 1(<25% cortical involvement) | 26/44(59.1%) | 24/46(52.2%) | 27/50(54%) | 22/45(48.9%) |  |
| Grade 2(25%-50% cortical involvement) | 0 | 0 | 0 | 0 |  |
| Grade 3(>50% cortical involvement) | 0 | 0 | 0 | 0 |  |
| **glomerular sclerosis proportion, median [IQR]** | 5.01(1.02,9.76) | 3.33(0,10) | 6.52(2.04,10.34) | 4.76(2.04, 10.5) | 0.746 |

IFTA, Interstitial Fibrosis and Tubular Atrophy; glomerular sclerosis, including ischemic sclerosis and segmental sclerosis.

**Supplementary Table S2. Cumulative Rituximab Exposure and Retreatment Details.**

| Characteristic | RTX (n =44) | RTX plus CsA (n = 50) |
| --- | --- | --- |
| **Standard initial induction (1g×2)，n%** | **2/44 (4.5%)** | **5/50(10%)** |
| **Additional RTX re-treatment induction, n%** | **42/44(95.5%)** | **45/50(90%)** |
| 1g | 3/44(6.8%) | 19/50(38%) |
| 2g | 26/44(59.1%) | 19/50(38%) |
| 3g | 13/44(29.5%) | 7/50(14%) |
| **Number of RTX infusions per patient** |  |  |
| Median (IQR) | 4(4，5) | 4(3，4) |
| **Cumulative RTX dose (initial induction+re-treatment ,g)** |  |  |
| Median (IQR) | 4(4，5) | 4(3，4) |

**Supplementary Table S3. Comparison of clinical remission rates stratified by baseline anti-PLA2R antibody titer across four treatment regimens: extremely high (>500RU/mL) and moderately high (150-500 RU /mL) subgroups**

| Characteristic | RTX | TAC | RTX plus CsA | CTX | P value |
| --- | --- | --- | --- | --- | --- |
| **Extremely high group(>500 RU/mL)** | **RTX (n = 6)** | **TAC (n=8)** | **RTX plus CsA (n = 13)** | **CTX (n = 6)** |  |
| Complete remission(CR) |  |  |  |  |  |
| 6months (n%) | 0 / 6 (0) | 0 /8 (0) | 1/ 13 (0) | 0 / 6 (0) | 1 |
| 12 months (n%) | 0 / 6 (0) | 0 /8 (0) | 1 / 13 (7.7) | 2 /6 (33.3) | 0.127 |
| 24 months (n%) | 0 / 6 (0) | 0 /8 (0) | 4 / 13 (30.8) | 2 /6 (33.3) | 0.143 |
| Partial remission (PR) |  |  |  |  |  |
| 6months (n%) | 0 / 6 (0) | 2 /8 (25) | 4 / 13 (30.8) | 0 /6 (0) | 0.231 |
| 12 months (n%) | 2 / 6 (33.3) | 5 /8 (62.5) | 6 / 13 (46.2) | 2 /6 (33.3) | 0.648 |
| 24 months (n%) | 2 / 6 (33.3) | 2 /8 (25) | 3 / 13 (23.1) | 4 /6 (66.7) | 0.276 |
| Total remission(TR) |  |  |  |  |  |
| 6months (n%) | 0 / 6 (0) | 2 /8 (25) | 4 / 13 (30.8) | 0 /6 (0) | 0.231 |
| 12 months (n%) | 2 / 6 (33.3) | 5 /8 (62.5) | 7 / 13 (53.8) | 4 /6 (66.7) | 0.648 |
| 24 months (n%) | **2 / 6 (33.3)*** | **2 /8 (25)*** | 7 / 13 (53.8) | 6 /6 (100) | **0.033*** |
| **Moderately high group(150-500 RU/mL)** | **RTX (n = 38)** | **TAC (n=38)** | **RTX plus CsA (n = 37)** | **CTX (n = 39)** |  |
| Complete remission(CR) |  |  |  |  |  |
| 6months (n%) | 1 / 38 (2.6) | 0/ 38 (0) | 3/ 37 (8.1) | 0 / 39 (0) | 0.093 |
| 12 months (n%) | **3 / 38 (7.9) *** | 11 /38 (28.9) | 14 / 37 (37.8) | 15 /39 (38.5) | **0.010*** |
| 24 months (n%) | 10 / 38 (26.3) | 15 /38 (39.5) | 19 / 37 (51.4) | 21 /39 (53.8) | 0.059 |
| Partial remission (PR) |  |  |  |  |  |
| 6months (n%) | **5 / 38 (13.2) *** | 16 /38 (42.1) | 16 / 37 (43.2) | 21 /39 (53.8) | **0.002*** |
| 12 months (n%) | 13 / 38 (34.2) | 16 /38 (42.1) | 14 / 37 (37.8) | 12 /39 (30.8) | 0.758 |
| 24 months (n%) | 12 / 38 (31.6) | 7 /38 (18.4) | 12 / 37 (32.4) | 6 /39 (15.4) | 0.186 |
| Total remission (TR) |  |  |  |  |  |
| 6months (n%) | **6 / 38 (15.8) **** | 16 /38 (42.1) | 19 / 37 (51.4) | 21 /39 (53.8) | **0.002*** |
| 12 months (n%) | **16 / 38 (42.1) *** | 27 /38 (71.1) | 28 / 37 (75.7) | 27 /39 (69.2) | **0.010*** |
| 24 months (n%) | 22 / 38 (57.9) | 22 /38 (57.9) * | **31 / 37 (83.8)** | 27 /39 (69.2) | 0.054 |

*P<0.05, **P<0.001, RTX plus CNI regimens as control.
